# Tandem duplication, circular permutation, molecular adaptation: how *Solanaceae *resist pests *via *inhibitors

**DOI:** 10.1186/1471-2105-9-S1-S22

**Published:** 2008-02-13

**Authors:** Lesheng Kong, Shoba Ranganathan

**Affiliations:** 1 Computational Biology Group, Temasek Life Sciences Laboratory, 1 Reseach Link National University of Singapore, Singapore 117604; 2 Department of Biochemistry, Yong Loo Lin School of Medicine, National University of Singapore, 8 Medical Drive, Singapore 117597; 3 Department of Chemistry and Biomolecular Sciences and Biotechnology Research Institute, Macquarie University, Sydney, NSW 2109, Australia

## Abstract

**Background:**

The Potato type II (Pot II) family of proteinase inhibitors plays critical roles in the defense system of plants from *Solanaceae *family against pests. To better understand the evolution of this family, we investigated the correlation between sequence and structural repeats within this family and the evolution and molecular adaptation of Pot II genes through computational analysis, using the putative ancestral domain sequence as the basic repeat unit.

**Results:**

Our analysis discovered the following interesting findings in Pot II family. (1) We classified the structural domains in Pot II family into three types (original repeat domain, circularly permuted domain, the two-chain domain) according to the existence of two linkers between the two domain components, which clearly show the circular permutation relationship between the original repeat domain and circularly permuted domain. (2) The permuted domains appear more stable than original repeat domain, from available structural information. Therefore, we proposed a multiple-repeat sequence is likely to adopt the permuted domain from contiguous sequence segments, with the N- and C-termini forming a single non-contiguous structural domain, linking the bracelet of tandem repeats. (3) The analysis of nonsynonymous/synonymous substitution rates ratio in Pot II domain revealed heterogeneous selective pressures among amino acid sites: the reactive site is under positive Darwinian selection (providing different specificity to target varieties of proteinases) while the cysteine scaffold is under purifying selection (essential for maintaining the fold). (4) For multi-repeat Pot II genes from *Nicotiana *genus, the proteolytic processing site is under positive Darwinian selection (which may improve the cleavage efficiency).

**Conclusion:**

This paper provides comprehensive analysis and characterization of Pot II family, and enlightens our understanding on the strategies (Gene and domain duplication, structural circular permutation and molecular adaptation) of *Solanaceae *plants for defending pathogenic attacks through the evolution of Pot II genes.

## Background

Members of potato type II proteinase inhibitor family (Pot II) are one of the major serine proteinase inhibitor families which are mainly found in higher plants from *Solanaceae *families [1]. The accumulations of Pot II inhibitors are always in response to stress, infection and wounding. They are one important measurement for plants to defense against predators or diseases. Intensive researches have been conducted on proteinase inhibitors (PIs) from this family. Interesting phenomena in Pot II family (such as tandem duplication, domain swapping and fold circular permutation [2,3]) make this family a good example to study gene evolution and protein folding. Members within this family have been identified with different numbers of tandem sequence repeat units (RUs), such as two [4], three [5], four [6], six [7], seven [8] and eight [9] RUs. Each RU can be characterized as a ~50-residue-long 8-cysteine polypeptide, which includes a reactive site targeting serine proteinases. The evolution of several members of this multi-domain family, at the gene duplication level, has been reported (as the Pin2 family [10]) in 2002. However, the complex correspondence between sequence repeats and their 3D structure and the molecular adaptation within this family has not been well investigated.

Several 3D structures of the Pot II family are known [1,2,11-15], belonging to the plant proteinase inhibitors family by SCOP (Structural Classification of Proteins) [16] fold family of plant proteinase inhibitors. The plant proteinase inhibitor family RUs adopts a variety of structural repeats, by circular permutation of the same fold [1,2,14]. Structures exhibited by naturally occurring proteins are single- or double-chain permutated domains composed of N- and C-terminal segments from sequence repeats. The engineered putative ancestral domain protein alone has a fold corresponding to the sequence repeat unit [2].

We have investigated the correlation between sequence and structural repeats within this family using sequence, structural and phylogenetic analyses, with the putative ancestral domain sequence as the basic repeat unit. Systematic analysis of Pot II family using bioinformatic approaches has revealed many interesting findings, of which the significant is the selection of the permuted structural domain as the preferred structural repeat unit, since it ensures the viability of proteinase inhibitory activity even as the native protein undergoes proteolytic cleavage.

## Results and discussion

### Protein 3D structures analysis of Pot II family

All the identified 3D structures of the Pot II family were classified into plant proteinase inhibitors family by SCOP [16]. Among these structures, only 1FYB and 1PJU are two-domain PIs while the rest have a single domain. All these structures have little secondary structure and are restrained principally by four disulphide bridges in each domain, and the main secondary structure in their folds is an anti-parallel 3-stranded β-sheet on the face opposite to the reactive site loop.

**Figure 1:**
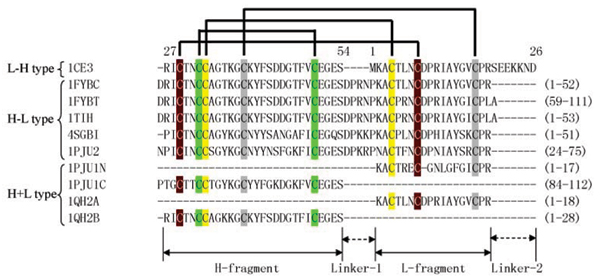
**Multiple sequence alignment of domains of all structures in the Pot II family.** The arrow marks out the positions of the reactive sites and the numbers refer to amino acid position. Pairs of cysteines forming disulfide bridges are linked by lines. Abbreviations used: 1FYB-C, chymotrypsin-specific domain of 1FYB (Domain I); 1FYB-T, trypsin-specific domain of 1FYB (Domain II); 1PJU-2, Domain II of 1PJU; 1PJU-1N, N-terminal segment of 1PJU (Domain I); 1PJU-1C, N-terminal segment of 1PJU (Domain I); 1QH2-A, chain A of 1QH2; 1QH2-B, chain B of 1QH2.

The sequence alignment of domains of the Pot II family structures (Figure 1) suggests that the sequences of all domains can mainly be divided into two parts, named here as the H- and L-fragments (for heavy and light fragments) connected by Linker-1 or Linker-2. In most structures, the L-fragment forms the reactive loop and one strand of the β-sheet, while the H-fragment forms a loop and two strands.

From Figure 1, clearly all the structures share the same disulfide connectivity although the combination of the H- and L-fragments is different. These domains can be divided into three types based the existence of two linkers (Linker-1 and Linker-2): (1) H-L type (H- and L-fragment joined by Linker-1): with structural examples, 4SGB-I, 1TIH, 1FYBC, 1FYBT and 1PJU2; (2) L-H type (L- and H-fragment linked by Linker-2): the engineered protein 1CE3; (3) H+L type (No Linker-1 or Linker-2 between two fragments): 1QH2 and 1PJU1.

**Figure 2:**
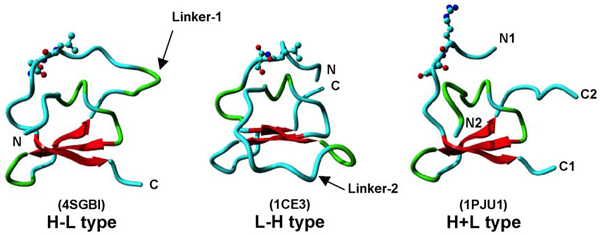
**Structural comparison of three types of Pot II PI topologies: H-L, L-H and H+L**. The structures are in ribbon representation, with the N- and C-termini marked and the reactive sites depicted in ball-and-stick mode. The β-strands are shown in red, with the linker regions marked.

The three structures shown in Figure 2 are actually the circular permutations of the same fold. All three topologies have the β-sheet and the functional proteinase inhibitory site conserved, although the intra-fragment connectivities are different. The H+L structure (1PJU1) can be considered the basic fold, with Linker-1 between C2 and N1 in 4SGB-I and Linker-2 between C1 and N2 in 1CE3. The existence of the H+L structure shows the viability of a two-chain protease inhibitor in this fold family.

For an individual single-domain Pot II protein, it could only have one topology from the three possible topologies depending on its primary sequence. But for a multi-RU Pot II protein, theoretically there are two possible domain organizations: (1) tandem repeat domain organization; (2) circularly permuted domain organization. For tandem repeat domain organization, the domains are arranged in beads-on-a-string way. Each domain is equivalent to the sequence repeat and adopts L-H topology. While for circularly permuted domain organization, the N- and C-termini are connected and formed one domain. In such a bracelet-like organization, the structural domains are not corresponding to the sequence repeats. The domain formed by N- and C-terminal sequences adopts H+L topology and other internal domains adopt H-L topology.

So the problem is: given a multi-RU Pot II protein, which kind of domain organization will it take? Based on the observation of the current data set, all experimentally determined multi-domain structures have circularly permuted two-domain organization (an H+L domain and an H-L domain). And most single-domain Pot II PIs (often derived from processing of multi-domain PIs) adopt the H-L type topology which also suggests that the multi-domain PIs have circularly permuted domain organization before they were processed. The only exception is 1CE3, which has only one RU in its primary sequence and thus can only adopt L-H topology, and moreover it is a engineered gene [2]. The abundance of H-L topology suggested it is more favourable by nature than L-H topology.

So the next question is: does H-L topology have advantage (e.g. more stable or better packing) over L-H topology? To evaluate the structure quality of different topologies and domain organization, we used several structure validation methods (WHATIF packing quality control [17], ERRAT [18] and ProQ [19]) to compare representative structures from each types. To facilitate the comparison, two 3-D models (named PI2t1 and PI2t2) of a two-RU PI, Potato Inhibitor II (PI-II) [Swiss-Prot: P01080], were built according to tandem repeat domain organization and circularly permutated domain organization, respectively.

The structure validation methods used in this study evaluate the structure quality of protein models from different aspects. WHATIF packing quality control is designed to test the proper packing of protein models by evaluating atomic contacts and calculating a contact quality index [17]. ERRAT detects incorrectly determined regions of protein models by analyzing the statistics of non-bonded interactions between different atom types [18]. The overall quality factor indicates the percentage of correctly determined regions in the protein models. ProQ predicts the quality of a protein model by using a neural-network-based method that based on a number of structural features [19] using two different measures, LGscore [20] and MaxSub [20]. For all the methods, higher value suggests better structural quality. Based on the results showed in Table 1, WHATIF packing quality control suggests that permuted structures and H-L type structures have better packing quality than tandem repeat and L-H type structures, especially for the fine packing quality control criteria. ERRAT and ProQ also recommend permuted structures and H-L type topology have better structure qualities and less problematic. Based on all above analyses, we believe that H-L type topology has better structure quality and is more favourable than L-H type topology. Therefore multi-domain Pot II proteins should tend to fold as H-L topology domains.

**Table 1:** **Quality comparison of representative structures using different structure validation methods**. The better scores were shown in bold.

**structures**	**Domain organization**	**Domain topology**	**WHATIF quality control**		**ERRAT**	**ProQ**	
					
			**Coarse**	**Fine**		**LGscore**	**MaxSub**
1PJU	Permuted 2D	H-L, H+L	**-1.587**	**-0.95**	**92.157**	**1.691**	**0.094**
PI2t1	Tandem 2D	L-H, L-H	-2.113	-4.60	57.282	1.416	0.072
PI2t2	Permuted 2D	H-L, H+L	**-1.540**	**-2.36**	**86.408**	**2.020**	**0.131**
1PJU-2	1D	H-L	**-1.554**	**-0.43**	**88.095**	0.918	**0.079**
1CE3	1D	L-H	-1.932	-3.43	47.862	0.088	-0.085
1QH2	1D	H+L	-2.198	-2.73	NA	0.197	-0.098

### The gene structure of Pot II family

Gene structures can potentially provide clues for the evolution of Pot II family. In 2002 Barta and colleagues reported that the conserved gene structure for this family being two exons separated by a 100–200 bp type I intron (phase 1) [10]. With more sequences and genomic data available, we resurveyed the gene structure and the genomic distribution of the Pot II family genes. We first collected exon/intron organization information for all available Pot II family members. TBLASTN searches were carried out with PI-II against the GenBank non-redundant database as well as the *Oryza sativa *genome and the assembled *Arabidopsis thaliana *genome from TIGR with the default parameters. All the significant hits (which contain the common eight-cysteine motif of Pot II genes) were combined, and only records that have complete coding sequence (CDS) information were retained. The final dataset contains 30 genes, and all of them come from plants. More specifically, most of them were from *Solanaceous *family species except one entry each from *Arabidopsis thaliana, Oryza sativa *and *Zea mays*. Only 13 entries from the 30 genes have intron information available. Among these 13 records, six are from *Solanum tuberosum*, four from *Lycopersicon esculentum *and one each from *Nicotiana tabacum, Oryza sativa *and *Arabidopsis thaliana*.

The distribution and chromosomal locations of Pot II genes can provide us the insights for the gene duplication history and mechanisms of Pot family. For plants, currently the whole genome sequence data is only available for *Arabidopsis thaliana *and *Oryza sativa*. The distribution of Pot II gene in the *A. thaliana *and *O. sativa *genome was investigated using TBLASTN searches. The assembled whole genome sequence for *A. thaliana *is available from TIGR *Arabidopsis thaliana *Database . The results show that there is only one copy of the Pot II gene (named after AT-PI) in the entire *A. thaliana *genome, with one RU [TIGR Arabidopsis thaliana Genome Annotation Database Locus: F28P5.12]. The putative Pot II gene in *O. sativa *(OS-PI) is from whole genome shotgun sequence [GenBank: NM_001057714] [21]. As with *A. thaliana*, rice has a single copy of the 1-RU Pot II gene. Since there is only one copy of Pot II gene in both *A. thaliana *and *O. sativa*, the current data cannot provide us more information about the chromosomal locations of duplicated Pot II genes.

We collected the exon and intron information for all records and investigated their gene structures with the assistance of the Xpro database [22]. Interestingly, all the records have similar gene structure including putative Pot II genes from *A. thaliana *and *O. sativa*. First of all, all the records have two exons. The first exon encodes a part of the signal peptide (12–17 residues). The second exon encodes the remaining part of the signal peptide (7–12 residues) and the mature polypeptide. There is no intron between the RUs in the genes of multi-RU sequences. Secondly, the splice phases for all records are conserved as phase 1. These results are consistent with the report by Barta *et al. *[10]. Moreover, we found that the splicing motif is also conserved and found to be GT...AG. The last nucleotide of the exon 1 and the first two nucleotides of exon 2 always encode a Gly residue. The conservation of exon/intron organization, splice phase, splice motif and Gly residues all confirm the homologous relationship between the identified Pot II family members. The same gene structure features are found in AT-PI and OS-PI, which are strongly indicative of these two are also members of the Pot II family. Furthermore, we find that in all the Pot II family members lacking intron information, there is a conserved Gly in a similar location in their signal peptides (data not shown). These records come from a range of species of the *Solanaceae *family, such as *Solanum americanum*, *Solanum nigrum*, *Nicotiana glutinosa, Nicotiana alata *and *Capsicum annuum*. These results confirmed that this Gly (formed by the boundaries of two exons) in signal peptide is also a conserved feature for Pot II family.

Both AT-PI and OS-PI have only one L-H type RU. Although more than ten single-domain PI proteins (such as PCI-1 [1]) have been reported, none of them was found to be the direct translation product of a single-RU gene. On the contrary, most of them are identical to a part of multiple-domain PI precursors, indicating that these single-domain PIs are proteolytic products of multiple-domain PIs. Considering the range of multiple-domain PIs found in *Solanacea*, gene duplication mechanism has been suggested to play an important role in the evolution of the Pot II family members, with the ancestral gene having only one RU [2,10]. The characteristics of AT-PI and OS-PI strongly support this hypothesis.

### Protein sequence analysis

We collected the protein sequences of all Pot II family members and putative Pot II PIs from the NCBI non-redundant protein database and dbEST database. After removing duplicates, 40 non-redundant protein sequences remained, with 95 RUs. We named the RUs according the following convention: Total_number_repeats-Accession-Species-RU_number. For example, PI3-IP22_LYCES-LE-R1 represents the first repeat unit (R1) of the 3-RU (PI3) protein, IP22_LYCES (Swiss-Prot names, accession numbers and GenBank accession numbers are used whenever possible.) from *Lycopersicon esculentum *(LE). (Abbreviations for all species used in this study are: AT, *Arabidopsis thaliana*; CA, *Capsicum annuum*; LE, *Lycopersicon esculentum; *LH, *Lycopersicon hirsutum*; MC, *Mesembryanthemum crystallinum*; MT, *Medicago truncatula*; NA, *Nicotiana alata*; NE, *Nicotiana attenuate*; NG, *Nicotiana glutinosa*; NT, *Nicotiana tabacum; *OS, *Oryza sativa*; SA, *Solanum americanum; *SH, *Sorghum halepense*; SM, *Solanum melongena; *SN, *Solanum nigrum*; SP, *Solanum phureja; *ST, *Solanum tuberosum*; ZM, *Zea mays*).

**Figure 3:**
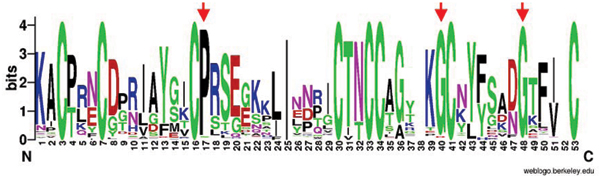
**Sequence Logo representation of the consensus sequence of all RUs from Pot II family**. The fully conserved residues are marked with asterisks ('*') and the highly conserved residues, by arrows.

From the consensus sequence of the multiple sequence alignment of the 95 Pot II family RUs, the 8 Cys residues are fully conserved. Besides these, other residues that are highly conserved are two Gly residues and a Pro residue (marked by arrows in Figure 3), probably having important roles in stabilizing the 3D structure of the protein.

The conservation degrees of the amino acid sites of Pot II RUs were estimated by a Maximum Likelihood method [23] and mapped to a reference 3D structure (PDB code: 1CE3) to identify functionally important regions by the program ConSurf [24].

**Figure 4:**
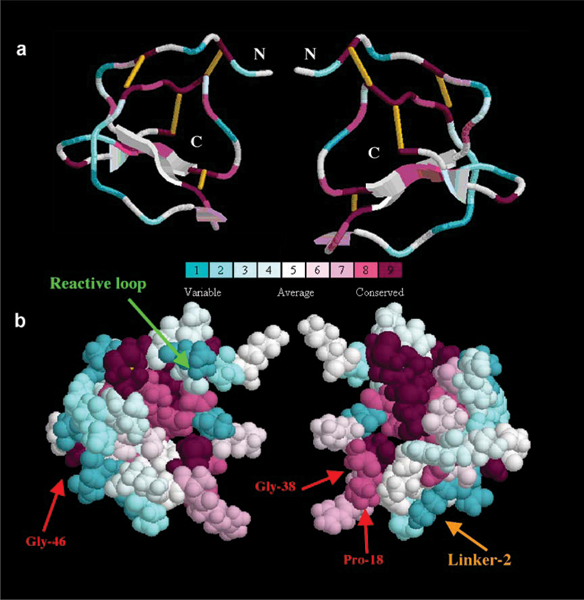
**Residue conservation analysis for the Pot II family RUs by ConSurf, mapped onto the structure, 1CE3**1CE3. Different views of the same structure were shown, rotated by 180°, in (a) ribbon and (b) CPK representations. Residues are shaded from cyan (highly variable) through white (moderate conservation) to purple (highly conserved).

Figure 4 shows that distinct regions in the RUs of Pot II PIs have very different conservation degrees. Besides the eight fully conserved cysteines as structural scaffold in the core region, a few highly conserved residues are also important for maintaining the fold, such as Pro-18, Gly-38 and Gly-46 (numbering according to 1CE3). The detailed analysis reveals that they belong to three β-turns, respectively. For example, the *i+3 *position of a type I β-turn is favored by a Gly residue, which is Gly-46, in 1CE3. Its phi-psi angle (80.3°, 63.7°) falls into the region that is not favored by other residues, and makes it hard to be replaced by other residues without distorting the fold. These 11 residues including the eight cysteines, are structurally important residues. Unlike most globular proteins, the reactive loop in this domain is highly variable. The variability of the reactive loop may allow the inhibitor to target varieties of proteinases efficiently. The two linker regions between the H- and the L-fragments (Figure 2), are also hypervariable which suggests that they are less critical for the functionality of the Pot II domain. 1CE3 has only linker region 2 (Linker-2, shown in Figure 4) and does not have the linker region 1, which is present in 4SGB-I (Figure 2).

### Phylogenetic analysis of Pot II family

**Figure 5:**
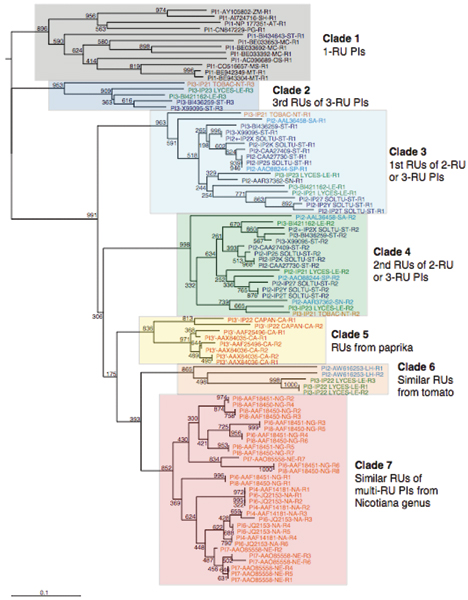
**Phylogenetic tree of Pot II PIs repeat units using NJ method.** The tree was bootstrapped for 1000 replicates. PIs from different species were colored into different colors. Green, tomato; dark blue, potato; red, paprika; orange, *Nicotiana *genus; blue, *Solanum *genus (except potato and tomato); black, non-*solanaceous *plants.

To investigate the evolution of Pot II family genes, the phylogenetic analyses were carried out using Neighbor-Joining (NJ), Maximum-Likelihood (ML) and Bayesian inference methods, respectively. In all three trees, the taxa can be clustered into seven clades by repeat number and species. All 1-RU PIs cluster into one group, and they are widely distributed in non-solanaceous plants. They are more distantly related to other members of the Pot II family and are more likely the ancestral single domain Pot II proteins. With only one RU, the sequence and the structural units are identical, with the L-H topology of 1CE3. We have defined all these single-domain PIs as outgroup (Clade 1) and re-rooted the trees. Figure 5 shows the NJ tree (see Additional File 1 for the ML tree and Additional File 2 for the MrBayes tree). The content of all clades are same in all the trees. The main difference of the three trees lies on the arrangement of clades. In all the trees, the basal branchings (the relative arrangements of clades) are relatively weakly supported by bootstrap values or posterior probability values. In NJ tree, Clade 2 (3rd RUs of 3-RU PIs), 3 (1st RUs of 2-RU or 3-RU PIs) and 4 (2nd RUs of 2-RU or 3-RU PIs) are clustered together (they are RUs from the same proteins), while in ML and MrBayes trees, these three clades are clustered with other clades. From current data, we cannot get better support information for the relationship between clades. In this study, our analysis does not depend on the relationship between clades and the selection among the three trees doesn't affect our analysis later. Here we showed the NJ tree and the other two trees are put into supplementary materials.

Figure 5 shows the inferred phylogenetic tree of 95 Pot II RUs. All RUs are clustered into seven clades, according to repeat number, species or total RU number. This clustering of RUs within each clade is strongly supported by the high bootstrap proportions (BP) where the relative positions between clades are tentative because their BP values are low. Clade 1 contains all (12 RUs) 1-RU Pot II PIs, which exist in a wide range of species and are more likely the ancient genes in Pot II family. The functionality or inhibitor activity of these genes is unknown because of the lack of experimental information. Clade 2 (5 RUs) comprises the third RUs of 3-RU PIs while Clade 3 (17 RUs) and 4 (17 RUs) consist of the first and second RUs of 2-RU and 3-RU PIs, respectively. Most of RUs in Clade 2, 3 and 4 are from *Solanum *genus plants. Clade 5 includes 8 RUs from paprika, and the sequence RUs in this clade are H-L type, which is different with RUs in all other members of Pot II family. Clade 6 (5 RUs) contains one 2-RU and one 3-RU PIs from *Solanum *genus. Clade 7 (31 RUs) includes 4-RU, 6-RU, 7-RU and 8-RU PIs from *Nicotiana *genus.

There are mainly three features observed in the conservation patterns (Figure 5).

(1) RUs with the same repeat numbers are most similar. The 2-RU and 3-RU PI from the *Solanum *genus (Clade 2, 3 and 4) contains 17 sequences, from 7 species with total 39 RUs, and is the largest group in this family. Here, the first RU clusters into one clade as do the second RU and the third RU. This suggests the duplication events happened before the speciation, although sequence similarity cannot be detected at the DNA sequence level between different repeats.

(2) Clade 5, 6, 7 contain repeats that are strikingly similar to each other within the same genes. The similarity is even clearly detectable at the DNA level (data not shown). Such pattern cannot be explained by purifying selection since the domain duplications usually loose the functional constraints and allow more mutations. The remarkable similarity suggests the existence of concerted evolution which usually can be resulted by unequal crossing over and gene conversion [25-27].

(3) In Clade 5, we have RUs from paprika that is very different to other members of the *Solanacae *species. Unlike all the other groups, the RUs of the Pot II inhibitor from *Capsicum annuum *are of the H-L type. The sequence repeat is thus identical to the structural repeat observed in potato and tomato and in *Nicotiana *(H-L type in Figure 1) and has no N- and C-terminal sequence segments, which form the "bracelet" link domain in other multi-RU PIs (H+L type in Figure 1). As each domain adopts the H-L domain topology, multiple-domain PIs from *Capsicum annuum *are likely to adopt tandem structural domains with a "beads-on-a-string" domain organization, which is different from all other multiple-domain PIs in Pot II family. Strong sequence similarity exists in this cluster at both protein and nucleotide sequence levels.

We believe that naturally isolated L-H type single-domain PIs can only be derived from single-RU genes, which are present in Clade 1, so far recognized in rice, maize, etc. Antcheva and colleagues reported the existence of a L-H type single-domain protein, PSI-1.2 [31,32]. But we cannot find any multi-domain protein (from NCBI nr) contains PSI-1.2 or any nucleotide sequence (from NCBI nt and dbEST) is corresponding to PSI-1.2. Therefore it is still uncertain that PSI-1.2 is derived from a Pot II gene with only one L-H type RU or it is the proteolytic processing product of a multi-domain gene.

### Analysis of selective pressure

Codon substitution models of were used to analyze Pot II genes to identify amino acid sites under diversifying selection. The models used the nonsynonymous/synonymous substitution rate ratio (ω = *d*_N_/*d*_S_) as an indicator of selection pressure and allowed the ratio to vary among sites. The ω ratio of a site <1 indicates that the nonsynonymous mutations at this site are deleterious and the site is under purifying selection while ω >1 suggests that the nonsynonymous mutations at this site are beneficial and the site should be under purifying selection.

Table 2 shows the parameters estimated under variable selective pressure among sites using the unrooted tree topology of Figure 5 without the out-group (PI1, single-RU Pot II genes). The average ω ratio ranges from 0.32 to 0.39 among all but the worst-fitting models. The Likelihood Ratio Test (LRT) statistics (Table 3) suggested the highly variable ω ratio among amino acid sites. For example, the null hypothesis model M0 (one ω ratio for all sites) is rejected by a big margin when compared with alternative hypothesis model M3 (discrete), which allows for three classes of sites with different ω ratios. The LRT statistic for this comparison is 235.12, which is much greater than the critical value 13.28 at 0.01% level from a χ^2 ^distribution with d.f. = 4. The discrete model (M3) suggests a small proportion of sites (p_2 _= 2.1%) under positive selection, with ω_2 _= 4.621. This model fits the data significantly better than M0 (one-ratio) or M1a (NearlyNeutral). Similarly, Model M8 (beta&ω) also suggests 2.1% of sites under diversifying selection with ω_1 _= 4.791. The LRT statistic for comparing null hypothesis model M7 (beta) and alternative hypothesis M8 (beta&ω) is 25.70, which is much greater than the critical value 9.21 at 0.01% level with the χ^2 ^distribution with d.f. = 2. M7 is thus rejected in favour of M8. In sum, among all the models tested, all models designed to detect positive selection sites (M2a, M3 and M8) were significantly better than their counterpart null hypothesis (M0, M1a and M7), which provide consistent evidence for the presence of heterogeneous selection pressure among amino acid sites within Pot II domains.

**Table 2:** Likelihood values and parameter estimates for Pot II genes

**Models**	***p***	***l***	**kappa**	***d*_*N*_ */d*_*S*_**	**Estimates of parameters**	**Positive Selected Site**
M0 (one-ratio)	1	-3281.13	1.706	0.262	ω = 0.262	None
M1a (NearlyNeutral)	2	-3219.57	1.986	0.551	p_0 _= 0.513, ω_0 _= 0.126 p_1 _= 0.487, ω_1 _= 1.000	Not Allowed
M2a (PostiveSelection)	4	-3201.55	2.045	0.714	p_0 _= 0.499, ω_0 _= 0.128 p_1 _= 0.480, ω_1 _= 1.000 p_2 _= 0.021, ω_2 _= 8.001	Site 5
M3 (discrete)	5	-3163.57	1.762	0.372	p_0 _= 0.363, ω_0 _= 0.041 p_1 _= 0.616, ω_1 _= 0.420 p_2 _= 0.021, ω_2 _= 4.621	Site 5
M7 (beta)	2	-3169.60	1.745	0.323	p = 0.525, q = 1.095	Not Allowed
M8 (beta&ω)	4	-3156.75	1.791	0.387	p_0 _= 0.979, (p_1 _= 0.021) p = 0.599, q = 1.450, w = 4.791	Site 5

**Table 3:** Likelihood Ratio Test Statistics (2Δ*l*)

**Comparison**	**2Δ*l***	**d.f.**	χ^2^_1%_	***p *value**
M0 (one-ratio) vs. M3 (discrete)	2 × [-3163.57-(-3281.13)]= 235.12	4	13.28	<0.0001
M1a (NearlyNeutral) vs. M2a (PostiveSelection)	2 × [-3201.55-(-3219.57)]= 36.04	2	9.21	<0.0001
M7 (beta) vs. M8 (beta&ω)	2 × [-3156.75-(-3169.60)]= 25.70	2	9.21	<0.0001

Furthermore, all models allowed positive selection (M2a, M3 and M8) converged to the same site, site 5. And site 5 had a high posterior probability (above the 99% level) of being in the positively selected class in all models allowed positive selection (M2a, M3 and M8).

Statistics analyses of variation of ω among sites provide strong evidence of the positive selection. Interestingly, the positively selected site 5 locates at P_1 _position of the reactive site of Pot II domains according the nomenclature of the Schechter and Berger [28]. For standard mechanism, canonical proteinaceous PIs of serine proteinases, the specificity of the inhibitors is determined, at least in part, by a single residue at the P_1 _position [29]. In Pot II PI structures, the P_1 _residue contribute the largest number of contacts [3]. Therefore, the hypervariability and positive selection of the P_1_residue in reactive site can be easily understood since they allow the Pot II inhibitors to provide inhibition activity to a wide range of proteinases, which help *Solanaceae *to fight against pathogenic attacks.

We also conducted clade-wise site-based analyses in selective pressure on Clade 3 (1^st ^RUs of 2-RU or 3-RU PIs), Clade 4 (2^nd ^RUs of 2-RU or 3-RU PIs) and Clade 7 (Similar RUs of multi-RU PIs from Nicotiana genus) in order to detect the short period of positive Darwinian selection within each clades.

**Figure 6:**
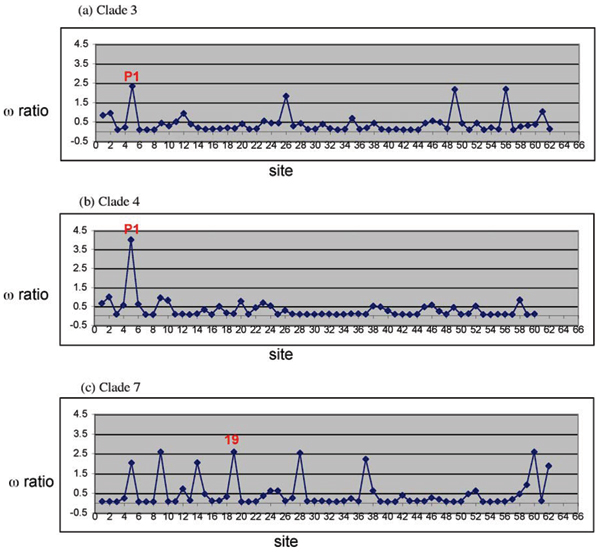
**Approximate posterior mean of the ω ratio for each site calculated under model M8**. The values were estimated by Bayes Empirical Bayes (BEB) method for (a) Clade 3 (1^st ^RUs of 2-RU or 3-RU PIs); (b) Clade 4 (2^nd ^RUs of 2-RU or 3-RU PIs); (c) Clade 7 (Similar RUs of multi-RU PIs from Nicotiana genus).

For all three clades, LRT tests support the existence of positive selected sites, but selective pressures among sites are quite different between Clade 3, 4 and Clade 7. We are interested in the variable selective pressure in different clades. For Clade 3, 4 and 7 separately, we plotted the approximate posterior mean of ω ratio at each site (Figure 6). Figure 6 shows that the majority of amino acid sites in Clade 3 and Clade 4 are under purifying or neutral selection while Clade 7 has more amino acid sites under positive selection. In Clade 3 and Clade 4, site 5 (P_1 _site of reactive loop) was identified as statistically significant positive selected sites by all models (M2a, M3 and M8), which is consistent with the previous analysis. While in Clade 7, all models support strong positive selection over site 19, which is the ending residue after the proteolytic processing removing the Linker 2 region (highly conserved linker "EEKKN" in multi-RU Pot II PIs from *Nicotiana *genus).

Such difference in selective pressure between Clade 3, 4 and Clade 7 may be due to the number of RUs. For two-domain Pot II PIs, the two domains can bind to two proteinases simultaneous without steric interference since the two binding sites are at opposite ends of two inhibitor domains (e.g. the bound form of TI-II) [12]. While for Pot II PIs with more than two domains, it becomes more and more difficult for each domain to bind a proteinase without steric hindrance. Heath and co-workers reported that the six-domain precursor NA-PI only has stoichiometry of 2.6 trypsin [30]. So the efficiency of proteolytic processing of multi-domain PIs may provide evolutionary advantages by performing better inhibition activity. This might be a possible explanation why in Clade 7 the residue on the cleavage sites is under positive selection.

## Conclusion

We have carried out systematic analysis of Pot II family on a significantly enlarged dataset comparing to the previous study by Barta and colleagues [10], using a wide range of bioinformatics analysis tools, leading to several interesting findings.

We classified the structural domains in Pot II family into three types (H-L, L-H and H+L) according to the existence of two linkers (Linker-1 and Linker-2) between the two domain components (H-fragment and L-fragment), which clearly show the circular permutation relationship between the H-L type and L-H type topologies.

Based on observed domain organization or all known sequences in Pot II family, there is a propensity in Pot II PIs domain's topology to adopt the H-L topology (representative structure being 4SGB-I). Given that the repeat unit for most multiple-RU Pot II PIs is of the L-H type, such PIs will therefore fold into contiguous permuted structural domains, linked by bracelet-like structures formed by the N- and C-terminal segments from the first and the last repeat units. For Pot II genes from paprika alone, the repeat unit is of the H-L type, so that multiple-domain PIs from paprika should adopt a simple tandem permuted domain architecture, with no linking bracelet structure, which is unique to the Pot II PI family.

The degree of conservation for each residue in the Pot II PIs repeat units was evaluated and mapped onto the molecular surface of the structure for the putative ancestral protein, 1CE3. The result shows that different regions of the protein sequences have very different mutation rates. Eight fully conserved cysteines form the scaffold in the protein core, with the reactive loop and linker region being highly variable. The rapid mutation of the reactive site is consistent with the PIs possessing the ability to adopt different specificities to target a wide range of proteinases. Three other highly conserved residues (two Gly's and a Pro) are located at structurally important sites β-turns and are thus critical for maintaining the Pot II domain.

Phylogenetic analysis shows that the repeat units cluster into several groups according to repeat number and species. The different similarities patterns between repeat units in genes suggest that in different species the duplication history and mechanism should be different. Two 3-repeat sequences from *Capsicum annuum *have evolved to tailor the sequence repeats to correspond with the structural repeats thus eliminating the bracelet link. The repeat unit for this group is a circular permutation of the ancestral domain, making this group the late entrant to the Pot II family.

The analysis of selective pressure in Pot II domain revealed heterogeneous selective pressures among amino acid sites: the reactive site is under positive selection (providing different specificity to target varieties of proteinases) while the cysteine scaffold is under purifying selection (essential for maintaining the fold). For multi-repeat Pot II genes from *Nicotiana *genus, the proteolytic processing site is under positive selection, which may be related to higher efficiency for cleavage.

Overall, our results unravel the strategies adopted by *Solanaceae *plants to fight against pests through the evolution of Pot II serine protease inhibitors. The duplications in both gene level and domain level enable rapid and efficient expression of Pot II genes. On the structure level, the multi-RU precursors can acquire circularly permutated structures that have a more stable and thermodynamic favourable folding. The molecular adaptation particularly the positive selection over reactive sites provides various inhibition activities targeting the broad range of pathogenic proteinases.

## Methods

### Collection of Pot II family members: structures, gene and protein sequences

To identify 3D structures in Pot II family, PSI-BLAST [33] was used to search against PDB [34] database with Potato Inhibitor II (PI-II) [Swiss-Prot: P01080] [4] sequence. The PDB codes for 7 retrieved structures are 4SGB[1], 1CE3[2], 1FYB[11], 1QH2[14], 1TIH[15], 1OYV[12], and 1PJU[13]. Among them, 1TIH, 1QH2 and 1FYB are from one or two domains (T1, C2 and C1-T1 domain, respectively) of NA-PI [7], a six-domain precursor from *Nicotiana alata*. The engineered single domain proteinase inhibitor, 1CE3, is the putative ancestral protein of Na-PI. For NMR structures where the PDB entry comprises multiple conformers, NMRCLUST [35] has been used to choose the representative structure. So the representative structures for 1CE3, 1FYB and 1TIH are model 9, model 4 and model 5, respectively. These monomers are named after 1CE3-9, 1FYB-4 and 1TIH-5. The structure of PCI-1, which comes from chain I of 4SGB, is named after 4SGB-I. 1OYV is a 2:1 complex of Subtilisin Carlsberg and the two-domain Tomato Inhibitor II (TI-II), and 1PJU is actually the unbound form of TI-II.

The gene structure of Pot II family may provide hints for evolution of the Pot II family. DNA sequences of Pot II genes were retrieved through a search of GenBank non-redundant database with TBLASTN using PI-II. Only complete DNA sequences were retrieved. TBLASTN searches were also performed against *Arabidopsis thaliana *and *Oryza sativa *genomes available from TIGR (The Institute for Genomic Research, ). The final dataset for Pot II genes was derived from the combination of the results of all these searches followed by redundancy removal and manual checking. The Accession numbers of 13 significant hits are AB110700, AK105387, AY007240, AY129402, L25128, M15186, NM_105864, U45450, X04118, X78275, Z12753, Z13992 and Z29537.

PSI-BLAST was used to search against NCBI non-redundant protein database to retrieve protein sequences of the Pot II family and TBLASTN was used to search against NCBI dbEST database [36]. The search results were combined with the collection of Pfam [37] entry Prot_inhib_II. Partial sequences and redundancies were removed. The final sequence dataset includes 40 protein sequences. The IDs (Swiss-Prot names, accession numbers and GenBank accession numbers are used whenever possible.) for these sequences were listed as follows: AAF14181, AAF18450, AAF18451, AAF25496, AAO85558, AAL36458, AAO88244, AAR37362, AAX84035, AAX84036, AC096689, AI724716, AY105802, AW616253, BE033392, BE033653, BE033692, BE942349, BE943304, BI421162, BI434643, BI436259, CAA27409, CAA27730, CN847229, CO516657, IP22_CAPAN, IP27_SOLTU, IP2Y_SOLTU, IP25_SOLTU, IP2K_SOLTU, IP2T_SOLTU, IP2X_SOLTU, IP21_LYCES, IP23_LYCES, IP22_LYCES, IP21_TOBAC, JQ2153, NP_177351 and X99095.

### Protein structure analysis

The alignments of 3D structures were performed using MULTI-GAFIT [38] and MALIGN3D algorithm in the MODELLER package [39]. The structures were displayed using RASMOL [40] and Swiss PDB Viewer [41]. Structural images were generated using YASARA (available from ). To evaluate the two kinds of domain organizations, MODELLER6v2 [39] was used to build homology models for each type. The two models are named after PI2t1 (tandem 2 domains, based on the template 1CE3) and PI2t2 (circularly permuted 2 domains, using 1PJU as the template). The structures of different types of topologies were compared to evaluate the structure qualities by using several structure validation methods, WHATIF Packing Quality Control [17], ProQ [19] and ERRAT [18].

### Gene structure analysis

The analysis of Pot II family gene structure (exon/inton boundary, organization and splicing phase) were facilitated by Xpro [22] and EMBOSS [42]. The *Arabidopsis thaliana *and *Oryza sativa *genomes were downloaded from TIGR.

### Protein sequence analysis

The sequences of Pot II proteins were extracted and then split into single Repeat Units (RUs) according to the putative ancestral domain sequence from 1CE3. The multiple sequence alignments were carried out with CLUSTAL_X [43] and followed by manual inspection and adjustment, to maximize the alignment of identical and similar residues and minimize the number of gaps. The consensus sequences were represented using Sequence Logos [44]. The degree of conservation of each amino acid was assessed by the maximum-likelihood method [23] and mapped onto the surface of the putative ancestral 3D structure (1CE3) using ConSurf [23].

### Phylogenetic analysis

Nucleotide sequences were retrieved from NCBI Entrez server and split into single RUs corresponding to putative ancestral domain sequence from 1CE3. The alignment of nucleotide sequences was facilitated by protal2dna server , based on the aligned amino acid sequences. Phylogenetic analyses using Neighbor-Joining (NJ), Maximum-Likelihood (ML) and Bayesian inference were carried out using PHYLIP 3.66 [45] and MrBayes 3.1 [46,47]. For Neighbor-Joining method analysis, NEIGHBOR program in PHYLIP was used to infer the phylogenetic tree [48]. DNAML program [49] in PHYLIP was used for ML analysis and the default parameters were used for the model setting. Bootstrap analysis (1,000 replicates) was done for both NJ and ML analysis to examine sampling error and local tree stability. For Bayesian inference, General time reversible model (GTR+I+G) was suggested as the best-fit model by MrModeltest 2.2 [50]. Bayesian analysis was carried out using MrBayes 3.1 with the following parameters: 2.5 million generations, 4by4 nucleotide substitution, sampled every 100 generations, with the consensus tree drawn using the last 20,000 trees. The trees were displayed using TreeView [51].

### Analyses of selective pressure

To examine the selective pressure acting on genes from Pot II family, we only used sequences from *Solanaceae *plants and excluded the single-RU Pot II genes since they are not well annotated and we are not sure whether they possess inhibition activity or not. The dataset included 83 RUs sequences from multi-RU Pot II genes after removing 12 single-RU genes. All the analyses were performed using the CODEML module of the PAML 3.15 package [52].

Codon-substitution Models of variable ω (nonsynonymous/synonymous substitution rates ratio) among sites were used to test for the existence of amino acid sites under positive selection (with ω > 1) and to identify these sites. We used several models (M0, M1a, M2a, M3, M7 and M8) recommended by Yang et al. [53,54]. Model M0 (one ratio) assumes invariable ω for all sites. Model M1a (NearlyNeutral) assumes two classes of sites in the protein: the conserved sites at which 0 < ω < 1 and the neutral sites at which ω = 1. In addition to the classes mentioned for M1a, the M2a Model (PositiveSelection) adds a third class of sites with ω as a free parameter, thus allowing for sites with ω > 1. Model M3 (discrete) uses a general discrete distribution with three site classes, with proportions (p_0_, p_1_, and p_2_) and the ω ratios (ω_0_, ω_1_, and ω_2_) estimated from the data. Model M7 (beta) assumes a beta distribution of ω (between 0 and 1) over sites depending on the parameters p and q. Finally, Model M8 (beta&ω) adds an extra class of sites to the beta (M7) model, therefore allowing ω values > 1. Among the above models, only Models M2a, M3, and M8 can detect sites under positive selection. From these models, Likelihood Ratio Test (LRT) can be done to test the positive selection hypothesis by comparing the simpler null hypothesis (M0, M1a and M7) with their more complex alternative models (M3, M2a and M8). All analyses were checked for convergence by performing the analysis with different starting ω values (0.3, 1 and 1.7). When the estimation of the parameters was finished, both naive empirical Bayes (NEB) [55,56] and Bayes empirical Bayes (BEB) [57] approaches were used to calculate the posterior probability for site classes. All statistics analyses were performed using the CODEML module in the PAML package [52].

Different Clades have distinct features and they may have different selective pressure over different amino acid sites. We conducted clade-wise site-based analyses in selective pressure on Clade 3 (1^st ^RUs of 2-RU or 3-RU PIs), Clade 4 (2^nd ^RUs of 2-RU or 3-RU PIs) and Clade 7 (Similar RUs of multi-RU PIs from Nicotiana genus). Other clades cannot be analyzed separately since they contain too few sequences.

## List of abbreviations

AT-PI: *Arabidopsis thaliana *Proteinase Inhibitor; BEB: Bayes Empirical Bayes; L1: Linker-1

L2: Linker-2; LRT: Likelihood Ratio Test; ML: Maximum-Likelihood; NJ: Neighbor-Joining

OS-PI: *Oryza sativa *Proteinase Inhibitor; PI: Proteinase Inhibitor; PI-II: Potato Inhibitor II; Pot II: Potato Type II proteinase inhibitor; RU: Repeat Unit.

## Competing interests

The authors declare that they have no competing interests.

## Authors' contributions

LK performed the data collection, bioinformatic analyses and drafted the manuscript. SR conceived of the study and participated in its design and coordination. Both authors have read and approved the final manuscript.

## Supplementary Material

Additional file 1 **Phylogenetic tree of Pot II PIs repeat units using Maximum-Likelihood method.** DNAML program in PHYLIP was used for ML analysis and the default parameters were used for the model setting. Bootstrap analysis was done for 1,000 replicates. Click here for file

Additional file 2 **Phylogenetic tree of Pot II PIs repeat units using Bayesian inference.** Bayesian analysis was carried out using MrBayes 3.1 with the following parameters: General time reversible model (GTR+I+G), 2.5 million generations, 4-by-4 nucleotide substitution, sampled every 100 generations, with the consensus tree drawn using the last 20,000 trees. Click here for file
