# Supplementary figures and images for: Tandem duplication, circular permutation, molecular adaptation: how Solanaceae resist pests via inhibitors
Source: BMC Bioinformatics. 2008 Feb 13;9(Suppl 1):S22. doi: 10.1186/1471-2105-9-S1-S22 (PMC2259423; doi:10.1186/1471-2105-9-S1-S22)

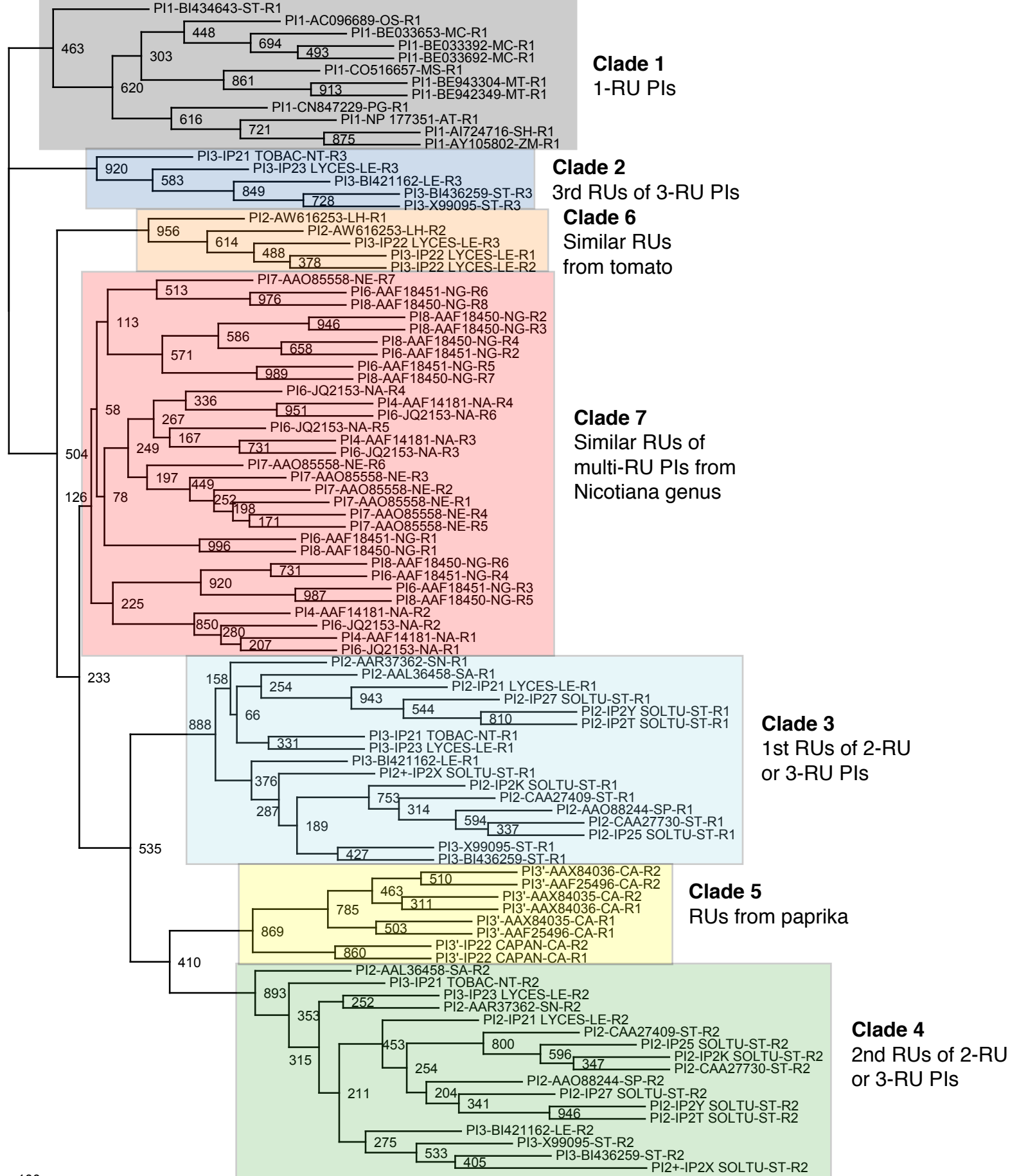

Supplement: Additional file 1 — Phylogenetic tree of Pot II PIs repeat units using Maximum-Likelihood method. DNAML program in PHYLIP was used for ML analysis and the default parameters were used for the model setting. Bootstrap analysis was done for 1,000 replicates. [file 1471-2105-9-S1-S22-S1.pdf]

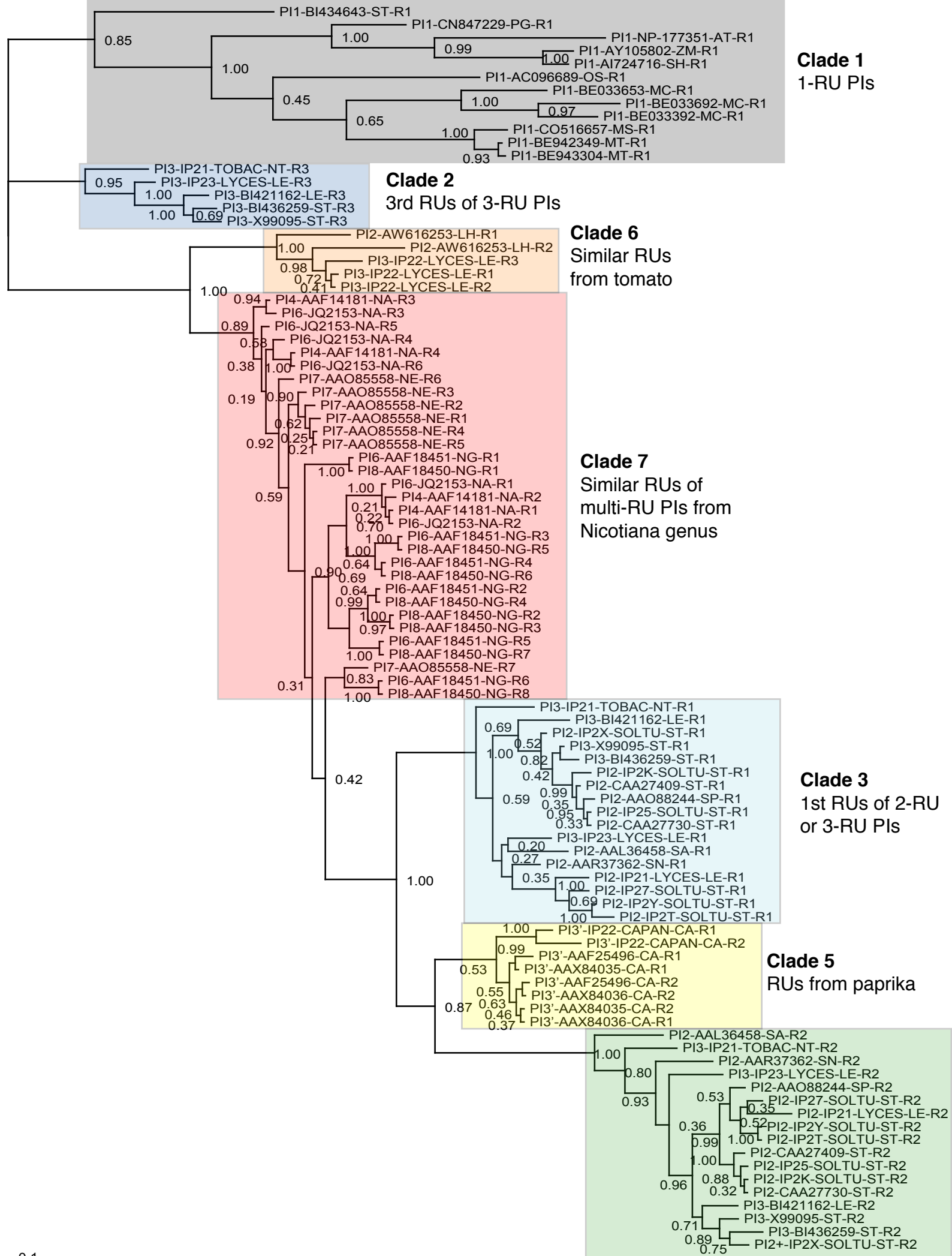

Supplement: Additional file 2 — Phylogenetic tree of Pot II PIs repeat units using Bayesian inference. Bayesian analysis was carried out using MrBayes 3.1 with the following parameters: General time reversible model (GTR+I+G), 2.5 million generations, 4-by-4 nucleotide substitution, sampled every 100 generations, with the consensus tree drawn using the last 20,000 trees. [file 1471-2105-9-S1-S22-S2.pdf]
